# Androgen receptor in breast cancer: The “5W” questions

**DOI:** 10.3389/fendo.2022.977331

**Published:** 2022-08-30

**Authors:** Sara Ravaioli, Roberta Maltoni, Barbara Pasculli, Paola Parrella, Anna Maria Giudetti, Daniele Vergara, Maria Maddalena Tumedei, Francesca Pirini, Sara Bravaccini

**Affiliations:** ^1^ IRCCS Istituto Romagnolo per lo Studio dei Tumori (IRST) “Dino Amadori”, Meldola, Italy; ^2^ Laboratorio di Oncologia, Fondazione IRCCS Casa Sollievo della Sofferenza, San Giovanni Rotondo, Foggia, Italy; ^3^ Department of Biological and Environmental Sciences and Technologies, University of Salento, Lecce, Italy

**Keywords:** anti-AR therapy, AR/ER ratio, AR biomarker, AR signaling, breast cancer

## Abstract

Androgen receptor (AR) is expressed in 60-70% of breast cancers (BCs) and the availability of anti-AR compounds, currently used for treating prostate cancer, paves the way to tackle specifically AR-positive BC patients. The prognostic and predictive role of AR in BC is a matter of debate, since the results from clinical trials are not striking, probably due to both technical and biological reasons. In this review, we aimed to highlight WHAT is AR, describing its structure and functions, WHAT to test and HOW to detect AR, WHERE AR should be tested (on primary tumor or metastasis) and WHY studying this fascinating hormone receptor, exploring and debating on its prognostic and predictive role. We considered AR and its ratio with other hormone receptors, analyzing also studies including patients with ductal carcinoma *in situ* and with early and advanced BC, as well. We also emphasized the effects that both other hormone receptors and the newly emerging androgen-inducible non coding RNAs may have on AR function in BC pathology and the putative implementation in the clinical setting. Moreover, we pointed out the latest results by clinical trials and we speculated about the use of anti-AR therapies in BC clinical practice.

## Background

Breast Cancer (BC) still remains the leading cause of cancer-related deaths for women, with an estimated 5-year prevalence in Europe in 2012 of about 1.8 million cases (1) and a staggering rate of about 7 million cases worldwide (2). BC has been recognized as an estrogen-sensitive disease. In particular, for BC patients the role of hormone receptor status is important to define the prognosis and to predict the response to endocrine therapy. Estrogen receptor (ER) expression can predict about 50-70% of tumor responses to anti-estrogen treatment (3–6). ER expression levels affect the BC relapse and ER positivity is commonly associated with better survival (7).

Since BC is highly heterogeneous, it is usually classified in five intrinsic molecular subtypes determined by a 50 gene-expression profile (PAM50), that have different clinical and biological characteristics affecting patient outcome (Luminal A, Luminal B, HER2-enriched, Basal-like, and Normal-like) (8). However, in the current clinical practice, this subtype classification is usually performed by immunohistochemistry (IHC) to detect the expression of Hormone Receptors (HR), human epidermal growth factor receptor 2 (HER2), and the proliferation marker Ki-67 (8). This evaluation has allowed a surrogate-subtype classification where cases are divided in Luminal A (HR+/HER2-/Ki-67 low), Luminal B/HER2-negative (HR+/HER2-/Ki-67 high), Luminal B/HER2-positive (HR+/HER2+/Ki-67 high), HER2-enriched (HR-/HER2+), and triple-negative BCs (TNBC) (HR-/HER-) (8).

The role of the androgen receptor (AR) in BC pathology is gaining clinical interest also in relation to the development of drugs that can modulate AR activity. Androgen receptors are expressed in 60%–90% of BCs, mainly in ER-positive tumors (about 70%) and in about 40% of triple negative tumors (8–10). However, its expression may vary depending on the cellular location (cytoplasmic and/or nuclear), the analytical methods used for the detection, the antibody used for IHC (11, 12), and the cut off used to establish AR positivity. Moreover, AR appears to exert different functions according to the BC subtype (9), *e.g.* in ER-positive BC AR may play an unfavorable prognostic role (13); in TNBC, AR could lead to a minor aggressive phenotype (14), but data from both clinical and preclinical trials are still controversial. However, AR is emerging as a new biomarker and potential therapeutic target in the treatment of BC patients. Recently, the availability of AR inhibitors used in prostate cancer (PCa) has advanced the possibility to use them in AR positive BC patients. Since initial findings do not appear striking, more clinical trials are required to set the proper treatment schedule and define the real clinical outcome with objective parameters. In case of proven efficacy, testing tumor tissues for AR would be recommended to determine potential benefit of AR-specific approaches to reduce risk of relapse. In this context much more attention should be paid to the standardization of analytical procedures and scoring systems for assessing AR expression, prior to upgrade the contemporary practice (15). In this review, we aim to explore and debate on the prognostic and predictive role of AR and the AR ratio with other hormone receptors, analyzing studies including patients with ductal carcinoma in *situ*, early and advanced BC, and outlining the recent findings on the association between AR and dysregulated microRNAs in the specific context of BC. Last, we describe also recent results that have been obtained from clinical trials and discuss key steps that are needed to translate anti-AR therapies into the clinic.

## What is AR: Structure and functions of the receptor

**Figure 1:**
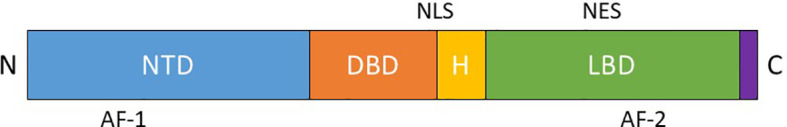
Functional domains of the androgen receptor: N-terminal domain (NTD), DNA binding domain (DBD), Ligand binding domain (LBD). (H – hinge region, AF-1 – transcriptional activating function 1, AF-2 – transcriptional activating function 2, NLS – nuclear localization signal, NES – nuclear export signal).

**Figure 2:**
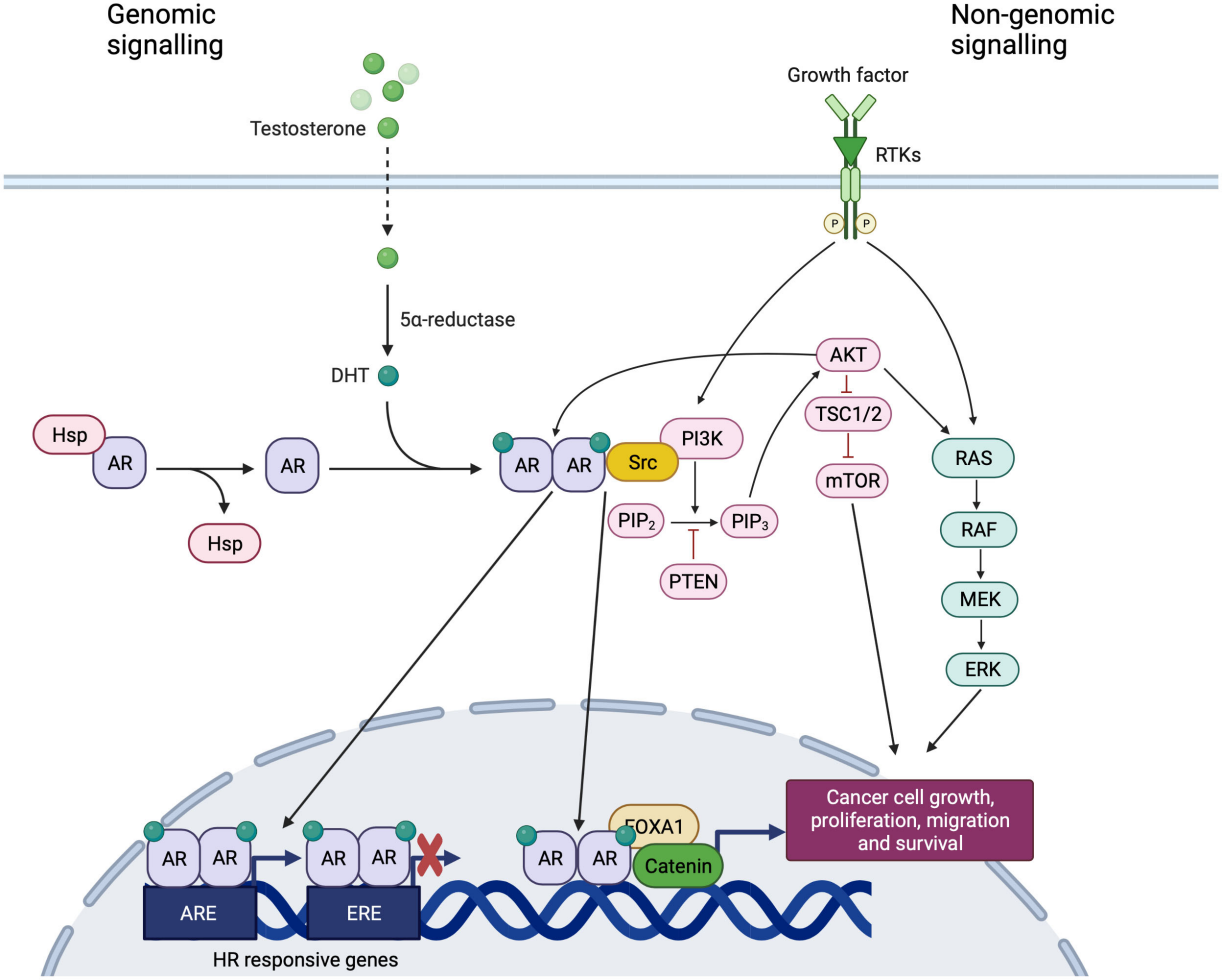
Genomic and non-genomic signaling of AR. Created with BioRender.com. In the absence of ligand, AR is located in the cytoplasm and is associated with heat-shock and other chaperone proteins. The binding of AR with androgens leads to the translocation of the complex to the nucleus, causes its dimerization and the binding to AREs, within classical target genes to modulate transcription. AR signaling exerts inhibitory effects on cell growth, interacting and binding to EREs and competing with ER. The DNA binding independent actions of AR are also commonly referred to in the literature as ‘non-genomic’ AR signaling with the downstream activation of alternative pathways, involving extracellular signal-regulated kinase (ERK), akt serine/threonine kinase (AKT) and mitogen- activated protein kinases (MAPK).

AR belongs to the steroid receptor superfamily and is made up of 919-aminoacids encoded by a 180 kb gene localized on human chromosome Xq11-12. The AR is expressed in a diverse range of tissues including bone, muscle, prostate, adipose tissue and the reproductive, cardiovascular, immune, neural and hematopoietic system (16). The receptor has three functional domains: a N-terminal domain (NTD, residues 1–555), containing activation functional regions; a DNA binding domain (DBD, residues 555–623) the most conserved region; and a carboxyl-terminal domain (CTD, residues 665–919) which includes the ligand-binding domain (LBD) (**Figure 1**). The DBD of all steroid hormone nuclear receptors consists of two zinc fingers that recognize specific palindromic consensus sequence 5′-GGTACAnnnTGTTCT-3′ called androgen response element (ARE) and facilitate the direct binding of AR to promoters and enhancers of AR-regulated genes, thereby allowing the functions to stimulate or repress the transcription. The DNA binding-dependent actions of AR are also commonly referred to in the literature as ‘genomic’, ‘classical’ or ‘canonical’ AR signaling. In the absence of ligand, AR is located in the cytoplasm and is associated with heat-shock and other chaperone proteins (**Figure 2**). The binding of AR with androgens leads to the translocation of the complex to the nucleus, causes its dimerization and the binding to AREs, within classical target genes to modulate transcription (**Figure 2**). The transcriptional activity is modulated by coregulators, able to enhance (coactivators) or repress (corepressors) the ability of AR to transactivate the target gene through chromatin remodeling and histone modifications (17) (**Figure 2**). The AR DNA binding domain can directly bind to DNA, but the sites are not readily available on compacted chromatin, tightly wound around nucleosomes. The chromatin must be “opened” by FOXA1, a pioneer factor with structural similarity to linker histones that is associated with AR at most AR binding sites (18). AR binding sites are also highly enriched for the GATA2 and OCT1 transcription factors, and GATA2 may have a pioneering function on a subset of genes (18). Many of the initially identified proteins recruited by AR, including the p160 steroid receptor coactivator proteins (SRC-1, 2 and 3), CBP, p300, and PCAF have lysine acetyltransferase activity and function as histone acetyltransferases (HATs) (18). Acetylation of lysines on histones may weaken their interaction with DNA; at some sites acetylation may also prevent modifications that repress gene expression. An additional important function for histone lysine acetylation is the recruitment of BRD4, which recruits the CDK9/cyclin T complex (positive transcription elongation factor b, P-TEFb) that phosphorylates RNA polymerase II to drive elongation. Interestingly, CDK9 can also directly associate with and phosphorylate AR. Changes in histone acetylation (mediated by HATs and histone deacetylases, HDACs) occur rapidly and were identified as the major posttranslational modifications mediating the transcription in response to hormone stimulation. In contrast, histone methylation on lysines was considered to modulate enhancer availability. However, with the discovery of multiple enzymes that can demethylate histones, it now appears that androgen stimulated methylation of histone and nonhistone proteins also contributes to gene activation. AR recruits and is coactivated by methyltransferases that may enhance the interaction between the AR NTD and LBD. In addition to its function as transcriptional activator, AR can also decrease the expression of several genes, binding and interfering with other transcription factors like SP1, RUNX2, JUN, and SMAD3, or β-catenin (18) (**Figure 2**). AR also may act more directly as a transcriptional repressor through an epigenetic mechanism by recruiting corepressors that mediate histone deacetylation, including ALIEN, DAX1, HEY, AES, PHB, and SHP, although the role of these corepressors in modulating specific AR regulated genes remains to be ascertained (18). In contrast to other steroid receptors, the androgen liganded AR can also interacts with the corepressors NCoR and SMRT that normally bind to the unliganded coactivator binding site in the LBD of nuclear receptors and are displaced after ligand binding. The interaction between AR and these corepressors probably occurs at a specific site of the NTD, and the downregulation of NCoR and SMRT can enhance the activity of the agonist liganded AR. An altered structure of the AR LBD generated by some AR antagonists may enhance NCoR and SMRT binding and contribute to antagonist activity, repressing the transcription of AR regulated genes (18).

The DNA binding independent actions of AR are also commonly referred to in the literature as ‘non-genomic’, ‘non-classical’ or ‘non-canonical’ AR signaling (19, 20) with the downstream activation of alternative pathways, involving extracellular signal-regulated kinase (ERK), akt serine/threonine kinase (AKT) and mitogen- activated protein kinases (MAPK) (21) (**Figure 2**). Indirect gene transrepression by AR binding can also occur through sequestration of transcription factors, such as the activator protein-1 (AP-1), that are normally required to upregulate target gene expression (21). Ligand-independent activation of AR *via* phosphorylation and/or interaction with co-activators promoted by a number of different growth factors has been widely demonstrated. For instance, IL-6, commonly expressed at high circulating levels in patients with different cancers, increases AR activity in a ligand-independent manner *via* the protein kinase A (PKA), PKC and MAPK pathways (18, 19) Similarly, an enhanced AR activation and nuclear localization is induced by epidermal growth factor (EGF) and insulin-like growth factor (IGF) and leads to the activation of MAPK signaling (22) (**Figure 2**). Specifically, low AR levels may have a scant transcriptional output, but consistently activate extranuclear signaling pathways (i.e., Src tyrosine kinase, or PI3K, or the filamin A-dependent pathway) leading to massive proliferation and invasiveness of target cells (23) (**Figure 2**).

### Crosstalk between hormone receptors and growth factors

The pathway of AR could promote or inhibit cell proliferation depending on the expression of other hormone receptors and their ligands. The interplay between AR, ER and their ligands is complicated by the possible conversion of androgens to estrogens. Patients with ER and AR-expressing tumors show a better outcome than those with ER-positive and AR-negative diseases (24, 25). One explanation could be the competition between AR and ER at the level of Estrogen Response Elements (EREs), that causes an impairment of ER-dependent gene transcription (26) (**Figure 2**). Thus, the binding of AR to EREs reduces the estrogen proliferative action and exerts anti-proliferative effects. Conversely, ER can bind to AREs leading to the opposite effect (27). This mechanism may explain the potential role of AR in the resistance to standard hormonal therapies (24). In fact, some studies have shown that AR and ER bind to the same DNA binding sites, demonstrating that AR could compete with ER-dependent transcription in ER+ BC (28). In HR positive BC, AR signaling exerts inhibitory effects on cell growth, interacting and binding to EREs and competing with ER and PgR (**Figure 2**). Conversely, in ER+/PgR– tumors, ERβ probably acts in a dominant negative manner, downregulating transcription of ERα target genes and the role of AR in the absence of PR is probably tumorigenic, enhancing ERα-mediated gene transcription (17).

In line with the documented pro-tumorigenic role of AR, De Amicis et al. showed that AR overexpression induces tamoxifen resistance in BC cell models. They hypothesized that

AR and ERα interact in the presence of Tamoxifen and are recruited to ER-responsive gene promoters, participating in the displacement of corepressor proteins, by recruiting coactivators, or even acting as coactivator itself (29). Cyclin D1 is a well-characterized target of ERα and its overexpression is a predictor of poor response to Tamoxifen in postmenopausal BC patients (29). AR overexpression could abrogate the ability of Tamoxifen to inhibit cyclin D1 levels, leading to a proliferative stimulus (29).

Indeed, the role of AR in ER-α-positive BC is controversial and a deeper knowledge of the crosstalk between HR is required. Hickey et al. using a clinically relevant panel of cell-line and patient-derived models, demonstrated that AR activation exerts potent antitumor activity in the resistance to endocrine and CDK4/6 inhibitors based therapies (30). Of note, AR agonists combined with standard-of-care agents enhanced the therapeutic response (30). In fact, the AR agonist activation altered the genomic distribution of ER and essential co-activators (p300, SRC-3), resulting in repression of ER-regulated cell cycle genes and upregulation of AR target genes, including known tumor suppressors. In addition, gene signature related to AR activity positively predicted the disease survival in ER+ BC patients (30).

Moreover, AR overexpression can activate the epidermal growth factor receptor (EGFR) pathway, promoting an agonist effect of tamoxifen on ER pathway and this mechanism could be blocked by a combination of enzalutamide and gefitinib (31). A crosstalk between AR pathway and HER2 pathway has been also reported (32, 33). In HER2-positive BC, AR regulates the expression of WNT7B that leads to the transfer of β-catenin into the nucleus (34). In the nucleus, the AR/β-catenin complex identifies the modulatory regions of HER3 and raises its transcription interacting with FOXA1 (**Figure 2**). HER3 and HER2 form a heterodimer that stimulates the expression of MYC and PI3K/AKT pathway, thereby resulting in boosting cell proliferation and tumor growth (34) (**Figure 2**). The synergism between AR and HER2 is further boosted by the mechanism i by which HER2 promotes AR transcription and leads to ERK activation that, in turn, regulates both HER2 and AR with a positive feedback loop (34). Moreover, in MCF-7 cells, it has been shown that ER and AR complexes can regulate c-ErbB2 signaling through c-Src engagement (35) (**Figure 2**).

### Androgen receptor regulation in breast cancer: The emerging role of non-coding RNAs

Non-coding RNAs (ncRNAs) are regulators of intracellular and intercellular signaling and control different cellular processes, cell proliferation, invasion, migration, apoptosis, and stemness. MicroRNAs (miRNA, miR) are 20-25 base pair long, single-stranded, non-coding RNAs which primarily bind to the 3’-untranslated region (3’-UTR) of messenger RNAs (mRNA) to suppress their transcription with the following reduction of target protein levels (36). Beside the well documented mechanism of action, the interaction between miRNAs and other regions, such as 5′ UTR, promoters and coding sequences (37),, and their ability to activate gene expression under particular conditions have also been reported (38). In addition, miRNAs may be shuttled between different subcellular compartments to control the rate of translation, even transcription (39). They could also be secreted in the extracellular space (40). lncRNAs are defined as RNAs longer than 200 nucleotides that are not translated into functional proteins. This definition includes a large and heterogeneous collection of transcripts that differ in their biogenesis and genomic origina. lncRNAs can modulate chromatin function, regulate the assembly and the function of nuclear bodies, alter the stability and the translation of cytoplasmic mRNAs and interfere with signaling pathways (41). In addition, lncRNAs can regulate mRNA expression by competing with miRNA in cytoplasm (42). Therefore, alterations affecting ncRNA expression have been linked to the pathogenesis of several human diseases, including cancer.

Several studies found and interplay among AR and ncRNAs in prostate cancer (43–45) and AR expression itself may be controlled by certain ncRNAs (45–47). Since controversial observations about AR oncogenic (48, 49) rather than tumor suppressor (50, 51) function in BC do exist, investigating the crosstalk between AR and ncRNAs may provide further insights into the role of AR signaling in AR+ BC, and unravel novel molecular features to take into account before planning an anti-AR therapy. In this context, the first effort focusing on BC is to be attributed to Nakano et al. (52), who identified miR-363 as an androgen-inducible miRNA upon treatment of Luminal A MCF-7 BC cells with low amounts of 5α-dihydrotestosterone (DHT). Interestingly, miR-363 was found to target a specific ligand-dependent co-activator of steroid receptors, i.e. IQWD1 (IQ motif and WD repeats-1), that is required to assemble a complex with AR preventing its proteasomal degradation (52). Subsequent investigations were extended to ER-/PR-/AR+ cancer cell models, and demonstrated that AR signaling, induced by DHT, directly upregulates let-7a expression, which in turn reduces the levels of its target oncogenes CMYC and KRAS impairing cell proliferation (53). This negative correlation was also confirmed by IHC in BC tissues (53). More importantly, a prognostic significance of AR and let-7a expression and their correlation was highlighted in invasive BC patients: high levels of AR and let-7a were correlated with and a small fraction of CD44^+^/CD24^-/low^ tumor-initiating cells expressing a stemness phenotype and patients with these features showed a better outcome (54).

Another crosstalk between miR-30a and AR further sustains an androgen-induced AR control of cell proliferation (55). In the original work, miR-30a was identified through a global miRNA expression profile performed on MDA-MB-453 cells treated with DHT, that found 43 up-regulated and 51 down−regulated miRNAs of whom miR-30a was reduced by androgen-induced AR signaling (55). The authors observed that DHT activated *in vitro* the AR downstream signaling, down-regulating miR-30a and preventing the inhibition it may exert on cell proliferation as tumor suppressor (55).

As alternative approach, Shi et al. (56) attempted to identify a distinct AR-associated miRNA pattern by comparing the miRNA expression profile of AR positive and AR negative BC cells. They obtained a signature of 153 differentially expressed miRNAs, with 52 upregulated and 101 downregulated in AR positive *versus* AR−negative cell lines. The most significant deregulated miRNAs, such as miR-143, miR-145, miR-31, and miR-181 were already known for playing a role in BC cell proliferation, invasion and drug−resistance. Furthermore, *in silico* target prediction and pathway enrichment analyses based on differentially expressed miRNA unraveled a putative association between AR and two key pathways of breast tumorigenesis: VEGF induced angiogenesis and mTOR associated tumor proliferation (56).

More recently, Bandini E. et al. have identified a new feedback loop involving AR and miR-9-5p, as androgen-inducible miRNA. Interestingly, they demonstrated that miR-9-5p may operate in AR+ BC by direct silencing of AR mRNA regardless of ER status, and even in presence of androgen agonists.

Moreover, although limited in size, a small cohort of formalin-fixed paraffin-embedded samples from paired normal/Luminal A and TNBC patients provided further evidences of a negative correlation between miR-9-5p and AR expression in tissues (57).

Other miRNAs that have emerged to undergo AR control, thus affecting cell growth and cellular morphology in BC are miR-21, whose androgen-induced reduction is a result of the recruitment of HDAC3 at *MIR21* gene promoter by AR (58), miR-100 and miR-125, which are inversely correlated with DHT-induced matrix metalloproteinase MMP13 (57), and miR-328-3p that increases upon DHT administration in MDA-MB-231 cells, acting as one of the mechanisms by which DHT reduces CD44 protein levels and limits cell motility and adhesion (59). To add further complexity to the androgen/miRNA/AR feedback loops likely involved in the BC pathology, Yang F et al. identified ARNILA, a long non coding RNA that acts as competing endogenous RNA through sequestering miR-204 and thereby favoring the expression of its target SOX4, which in turn promotes epithelial−mesenchymal transition (EMT), invasion and metastasis in TNBC. In AR-positive tumors, the lncRNA ARNILA expression was apparently suppressed by AR at transcription level, leading to increased release of miR-204 downregulating SOX4. Reciprocally, in AR negative BC, activated ARNILA promoted Sox4 expression by competitively binding to miR-204 (60).

Collectively, the data summarized in **Table 1** and new advisable investigations may improve the understanding of both biological involvement and clinical relevance of AR in BC, and encourage a careful examination of the therapeutic potential of an AR-based approach in potentiating the effectiveness of anti-estrogen adjuvant therapies or designing new therapies for estrogen-insensitive neoplasms.

**Table 1:** Putative AR Deregulated miRNAs and associated cancer hallmarks in breast cancers.

microRNA	AR effect on miRNA	Cancer Hallmark	Reference
**miR-363**	Androgen-inducible miRNA upon treatment with low amounts of DHT	Prevents AR proteins degradation.	(49)
**Let-7**	Up-regulation by AR activated signaling	Reduces levels of MYC and KRAS impairing cell proliferation	(50)
**miR-30a**	Down-regulated by AR activating signaling	Increase cell proliferation	(52)
**miR-21**	Down-regulated by AR signaling by recruiting HDAC3 at MIR21 gene promoter	Cell growth	(54)
**miR-100 mIR-125**	Inversely correlated with DHT induced MMP13	Tumor angiogenesis.	(55)
**miR-328-3p**	Up-regulation by AR activated signaling	Reduces CD44 protein levels affecting cell motility and adhesion	(56)
**miR-204**	AR suppression of ARNILLA and consequent miRNA sequestering	EMT promotion by increased expression of the target SOX4	(58)
**miR9-5p**	AR inducible miRNA	Regulation of AR expression	(57)

## How to test AR

**Figure 3:**
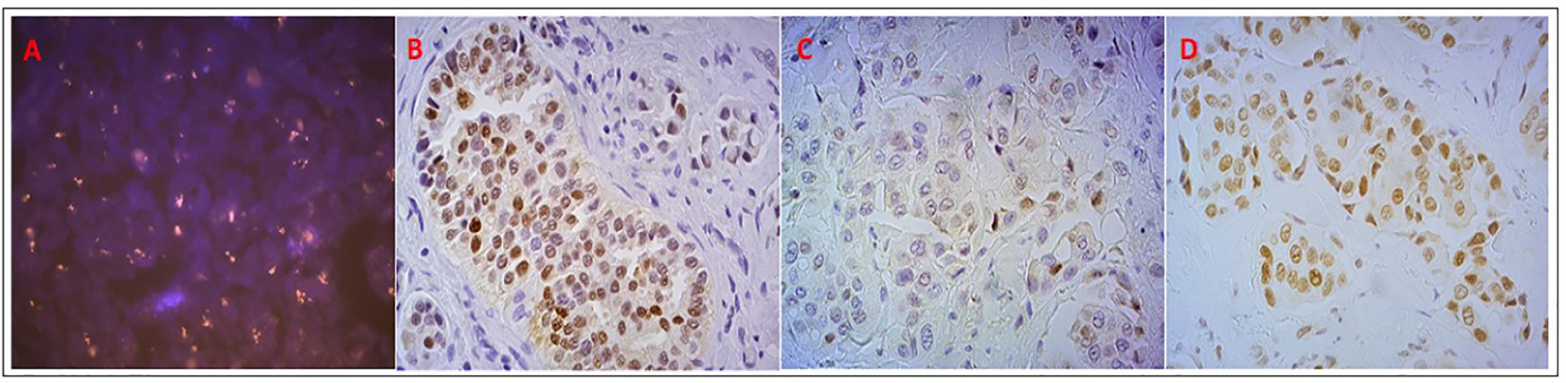
Analysis of AR status in the patient who had stable disease with DHEA: **(A)** AR copy number, evaluated by FISH, showing clusters of orange signals; **(B)** AR positive nuclear expression by IHC; **(C)** AR pSer210-213 positive but weak nuclear and cytoplasmic expression; **(D)** AR pSer650 positive nuclear expression.

After ligand binding, AR translocates into the nucleus inducing AR responsive gene transcription. When the levels of androgens decrease, the unliganded AR moves from the nucleus to the cytoplasm, where it is transcriptionally inactive. Tissue-based methodologies permit to study AR and its localization at cellular level, both in tumor cells and in the surrounding stroma (61). It is well established for BC characterization that the hormone receptor status assessment is done by IHC, the cheapest and fastest method that can be performed routinely in all laboratories also for the *in situ* evaluation of AR expression. Despite this consolidated method the immunohistochemical analysis of AR expression still presents some gaps, such as the different cut offs proposed to classify AR-positive cases (i.e. 0% or 10% of immunopositive tumor cells). *H* score was also proposed to define AR positivity, a semiquantitative parameter that considers both the staining intensity and the percentage of positive cells (62). Other methods have been used to assess different hallmarks of AR in tissues, such as the fluorescence *in situ* hybridization (FISH) to analyze the gene copy number (**Figure 3**), sequencing or PCR-based technologies to assess AR mutations, and gene expression analyses to evaluate AR transcript (61, 63).

Sequencing and functional analyses have revealed a membrane-associated form of AR present in MCF-7 cells and in T47D cells (64) and identified in the MDA-MB-453 cells (ER-) the AR-Q865H variant, harboring a mutation in the AR LBD, with reduced sensitivity to DHT and indicator of poor response to AR antagonists (65).

There is an increased need of biomarker assessments by non-invasive methods and new approaches to test AR by liquid biopsy have been developed for this purpose. Several studies in PCa have evaluated AR in serum, plasma or urine, demonstrating a correlation between copy number changes, mutations and splice variants with diagnosis, prognosis, tumor evolution and outcome (66, 67). In BC only few studies have been conducted evaluating AR in liquid biopsy, and in particular in circulating tumor cells (CTCs). For instance, the presence and the expression of AR-v7 splice variant, which lacks the LBD, detected on CTCs seems to be related to an increased number of bone metastasis (68). Given the evidences in PCa and its similarity with BC in terms of hormone dependency, the detection of AR-v7 in CTCs might represent a potential predictive marker for anti-AR treatment in BC (61). Additionally, AR45 represents another splice variants expressed in MDA-MB-231 and MDA-MB-453 that inhibits AR functions (66).

Recently, in metastatic BC, AR transcript was found in CTCs in 31% of samples and 58% of matched CTCs and primary tumor samples of different BC subtypes showed a discordance in terms of AR status evaluated as transcript, concluding that the determination of AR expression in CTCs could help to select metastatic BC patients potentially eligible for AR inhibitors (69). In sum, the detection of AR alterations by liquid biopsy in the context of BC requires further advances. Despite several assays based on gene expression profile are emerging (8, 63), the best and most commonly used method to assess AR expression remains the IHC performed on tissue samples. Nevertheless, a rigorous IHC standardization is needed to homogenously evaluate the results from clinical trials.

## Where AR should be tested: On primary tumor or metastasis

Only few studies have evaluated AR expression in matched primary tumors and metastases (70, 71). Kraby and colleagues reported discordant data on AR expression between primary tumor and correspondent lymph node metastases, observed in 21.4% of cases and often associated with a switch in AR status from negative primary tumor to positive axillary lymph node metastasis (72). Some authors observed that hormone receptor status (ER and PgR) may change several times over the course of the disease and these changes might be associated with prognostic worsening. Hence, they suggest to repeat the hormone receptor determination in metastatic BC patients (73). In this context, our group has highlighted an overall concordance greater than 64% in AR status evaluated by IHC between primary tumor and metastasis, eve using two different cut off values (1% and 10%) (74). However, since AR status may differ between primary tumor and matched metastases AR testing should be carried out in both specimens to help patient selection for anti-AR therapy (74). Additionally, we found that the difference in terms of AR positivity between primary and metastatic lesions was not due to the timing between samplings, suggesting that AR should be evaluated in all the biological material available for each patient (74).

Overall several studies suggest that although there is some stability of the intrinsic subtype, approximately 40% of the tumors will change subtype from the primary to the metastatic/recurrent tumor, highlighting the need to biopsy metastatic disease and to analyze its molecular profile to better understand the clinical and biological evolution (75). Nowadays the majority of the biological changes occurring during BC metastatic progression is largely unknown but they could be driven by the therapeutic pressure and they could be the results of tumor evolution and/or acquisition of estrogen-independency (75).

## Why studying AR

### AR prognostic role in DCIS

Although several studies made an effort to establish the prognostic role of AR in invasive BC, little has been done for ductal carcinoma *in situ* of the breast (DCIS). Recently, it has been reported that it is important to test AR by IHC to evaluate the utility of AR antagonists for chemoprevention in patients with AR+ and ER- DCIS (76). Patients with breast *in situ* tumors are routinely treated with surgery and radiotherapy (and Tamoxifen in some cases). In this context, the prognostic and predictive role of specific markers, including AR, for clinical outcome in this population was investigated by Ravaioli et al. (77, 78). In these retrospective studies, series of matched DCIS relapsed and non-relapsed cases, treated with quadrantectomy and quadrantectomy plus radiotherapy were analyzed, highlighting that AR and AR/ER ratio play an unfavorable prognostic role independently of the treatment. Most samples (91.7%) were AR-positive and the expression was significantly higher in the relapsed cases. AR expression was seen more frequently in high grade DCIS, and in the histological subtype with a solid growth pattern and apocrine features. Of the 78 AR positive cases, 21 (27%) were ER negative. It was demonstrated that the AR/ER ratio was statistically higher in relapsed patients of both case series, independently of the treatment, with high AUC values (92% and 80%), in patients treated with surgery and surgery plus radiotherapy, respectively. Taken together these data suggested that the hormone receptor expressions together with AR, could be important prognostic markers able to increase the accuracy in terms of relapse prediction for patients with DCIS (77–79).

### AR prognostic role in invasive breast cancer

Expression of AR was more frequently seen in luminal BC than in basal tumors, with the highest levels observed in Luminal A, while the lowest levels were observed in HER2-positive and TNBC (17, 80–83). Collins et al. reported that AR is most commonly expressed in Luminal A and B invasive BC and it is present in approximately one-third of basal-like cancers (12).

Some authors reported no association between AR expression and disease free survival (DFS) in ER expressing tumors (13, 84, 85), while ER remained an independent prognostic marker for patients undergoing endocrine therapy (13, 84, 85). However, Cochrane et al, reported that AR had a prognostic role in co-expression with other hormone receptors, while for others its prognostic value seems to be independent from the expression of the hormone receptors (10, 24). In particular, these authors performed a systematic review to study the association between AR expression and survival in women with early BC, calculating the odds ratios OR weighted and pooled in a meta-analysis with Mantel–Haenszel random-effect modeling.

In line with this finding, Kraby and colleagues demonstrated an independent favorable prognostic role of AR, in particular for grade 3 and Luminal A BCs (72).

### The importance of the ratio between AR and other hormone receptors

AR seems to play different roles depending on BC subtypes and in relation to ER expression. In ER negative BCs, AR expression does not have a clear prognostic effect (10), but it can predict response to AR inhibitors (15, 86). In about 80% of ER-positive BCs AR is expressed and its coexpression of hormone receptors is associated with a better prognosis and low grade tumors (80, 87, 88). The crosstalk between AR and ER in human cells of breast and prostate is well established and it is exerted at the level of EREs but also at non-genomic levels, involving Src tyrosine kinase and EGFR (32, 35, 89). Some therapeutic approaches have been proposed to target this complex crosstalk, balanced by different coregulators of different pathways (90). Cochrane et al. (24) demonstrated that AR nuclear expression in relation with ER in primary tumors predicts the benefit from adjuvant tamoxifen, on the basis of previous findings reporting that AR expression decreases in neo-adjuvant endocrine therapy responsive tumors (91, 92). The assessed prognostic and predictive value of AR/ER ratio in patients with primary HR+/HER2- BC treated with Tamoxifen (24) suggested that this marker could be useful for prognostic classification of luminal cancers. However, there are data about the role of AR/ER ratio as unfavorable prognostic marker only in invasive primary tumor of early BC patients and different cut off values have been used (13, 24). Similarly, we previously highlighted the unfavorable prognostic role of the AR/ER ratio in patients with *in situ* ductal carcinoma, independently of treatment (77, 78). About invasive luminal cancers, we found a potential role of AR/PgR ratio > 0.96 in predicting the efficacy of first-line endocrine treatment in HR+ advanced BC (81). In addition, we found in another luminal patient cohort, analyzing both primary tumor and metastasis, that a high AR/ER ratio observed in both samples was associated with a better prognosis, while an AR/PgR ≥1.54 was significantly correlated with worse outcome (HR: 2.27 95%CI: 1.30-3.97; p = 0.004), suggesting its possible role as an additional risk-stratification marker in luminal BC (83).

In conclusion, a prospective study is needed to better clarify the role of the ratio between AR and other hormone receptors in different BC settings.

## Therapeutic targeting of AR

### Androgens in breast cancer: AR agonists

The sex hormones testosterone and DHT acting *via* AR are the androgens required for the development of the reproductive system and secondary sexual characteristics (93). Testosterone can be converted to its active form DHT and to estradiol by 5α reductase and aromatase, respectively (94). The circulating androgens are dehydroepiandrosterone-sulfate (DHEA-S), dehydro-androstenedione (DHEA), secreted by adrenal glands, testosterone and androstenedione (A4), produced by ovaries (95, 96). They all play key roles in the functionality of reproductive system, muscle growth and prevention of bone loss. In pre- and post-menopausal women the levels of circulating androgens undergo many changes: testosterone start to decline before the menopause and A4 and DHEA levels decrease throughout post-menopause, as consequence of the reduced functionality of the ovaries. However, this change is less drastic than the decrease in levels of estrogen and progesterone (97).

Androgens have different effects among BC subtypes and AR agonists have been considered for a possible therapeutic strategy in BC (15, 98–100). In different BC models, they could have antiproliferative effects in co-expression with ER (101, 102) and pro-proliferative in ER absence (103, 104). In the former context, the AR promotes cell proliferation by acting at different levels indicating a potential unfavorable role of AR in HR+ BC. Conversely in TNBC, AR may have both favorable prognostic and predictive value, since increasing evidence suggests that AR positive TNBC patients may respond to AR targeting agents (14).

DHEA is transformed into sex hormones within peripheral target tissues (96, 105–107), as well as in BC cells where preclinical evidences of DHEA antitumor activity are reported (108–110). In this context, a phase II prospective clinical study was conducted, evaluating the safety and the activity of DHEA combined with AI in two AR positive metastatic BC patient cohorts: one ER-positive and one TN (111). The administration of an aromatase inhibitor (AI) prevents DHEA conversion into estrogens and favors its conversion into androgens. The treatment was considered safe, despite it showed a poor efficacy, possibly due to heavy pretreatment of the patients that caused a reduced hormone sensitivity, and maybe due also to the variability in adrenal function (112). The AR gene amplification present in the only patient who showed a prolonged clinical benefit was intriguing, prompting to hypothesize the potential value of AR gene amplification as a predictive biomarker of response to AR agonists in BC (**Figure 3**). Similarly, the role of phosphorylated AR remains to be ascertained (**Figure 3**) (111). However, this study was limited by the small number of patients considered and the low rate of clinical benefit and no definitive conclusions could be drawn.

Enobosarm (GTx-024) is a non-steroidal selective androgen receptor modulator (SARM) that has demonstrated preclinical and clinical activity in AR positive BC (113). Palmieri and colleagues tested the efficacy of enobosarm in a phase 2 trial including 136 postmenopausal patients with metastatic or locally advanced AR+/ER+ BC. AR-positive tumors were defined in presence of >10% nucleic AR expression. Patients were randomized to receive two different doses of enobosarm (9 or 18 mg oral daily). The clinical benefit correlated with the % of AR expression: with the cut off of AR>40% the best results in terms of efficacy were observed (113) (**Table 2**). Recently, another phase 2 trial tested safety and efficacy of the combination of enobosarm and pembrolizumab in AR+ metastatic TNBC, heavily treated without PD-L1 preselection (118). Although the trial was stopped early because of the withdrawal of GTx-024 drug supply, the combination of enobosarm and pembrolizumab was well tolerated, with a modest clinical benefit rate of 25% at 16 weeks (118).

**Table 2:** Ongoing clinical trials including AR agonists and antagonists with results.

Trial ID	Aim	Setting	phase	status	Results
**NCT02463032**	to determine the efficacy and safety of **enobosarm**	Metastatic or Locally Advanced ER+/AR+ BC (Postmenopausal)	II	Completed	Enobosarm treatment was well tolerated with significant positive effects on quality of life measurements. A higher % AR staining correlates with a greater antitumor activity (111).
**NCT02971761**	To determine the side effects the efficacy of pembrolizumab and **enobosarm**	AR+ metastatic TNBC	III	Active, not recruiting	The combination treatment was well tolerated, with a modest clinical benefit rate of 25% at 16 weeks in heavily pretreated AR+ TNBC without preselected PD-L1 (114).
**NCT01381874**	to assess the safety and efficacy of **abiraterone acetate (AA)**+ prednisone and AA+ prednisone +exemestane, each compared with oral exemestane alone	postmenopausal women with ER+ metastatic BC that has relapsed after treatment with letrozole or anastrozole	II	Completed	Adding AA to E in NSAI-pretreated ER+ MBC patients did not improve PFS compared with treatment with E. An AA-induced progesterone increase may have contributed to this lack of clinical activity (115).
**NCT03004534**	to evaluate molecular alterations in human breast cancer tissue following short-term exposure to **darolutamide**	Early BC	I	Completed	The authors evaluated how the treatment may change the genes or proteins in BC cells and its safety and the way it is tolerated by subjects (116).
**NCT01889238**	to determine the safety and the efficacy of **enzalutamide**	AR+ advanced TNBC	II	Active, not recruiting	Enzalutamide demonstrated clinical activity and was well tolerated in patients with advanced AR+ TNBC (124).
**NCT02091960**	to evaluate the efficacy of **enzalutamide** with trastuzumab	HER2+ AR+ metastatic or locally advanced BC	II	Active, not recruiting	Enzalutamide+ trastuzumab was well tolerated, and a subset of patients in this heavily pretreated population had durable disease control (124).
**NCT02007512**	to determine if **enzalutamide** given in combination with exemestane is safe and effective	ER /PR+ HER2- advanced BC	II	Active, not recruiting	Enzalutamide with exemestane was well tolerated. PFS was not improved in an unselected population, ET-naïve patients with high AR and low ESR1 mRNA levels may benefit from enzalutamide+exemestane (117).

Anti-AR agents are indicated in bold.

### A lesson from prostate cancer: AR antagonists

Prostate cancer growth and progression are sustained by AR signalling, hence androgen deprivation therapy is the gold standard treatment in PCa. AR upregulation is the most common event underlying the progression from hormone sensitive to castration-resistant PCa. AR overexpression can occur caused by different mechanisms, including mutations, amplifications, gene rearrangements, that produce truncated AR variants (119). Several AR targeted therapies, like abiraterone, enzalutamide, apalutamide and more recently darolutamide (120) have been developed and tested in AR+TNBC patients. Much of the information about the role of AR derives from studies conducted in the context of TNBC, an aggressive disease with poorer outcomes than other BC subtypes. Gene expression profiling of TNBC have identified the luminal androgen receptor (LAR) subtype that is dependent on AR signaling and accounts for about 22% of all TNBCs (121). Despite, LAR TNBC patients have been shown to have a better prognosis than those that are AR-negative, a lower pathologic complete response (pCR) rate was seen in patients with positive TNBC undergone neoadjuvant therapy (121).

Abiraterone is not an AR antagonist, since it is a selective inhibitor of the cytochrome P450 involved in androgens biosynthesis. It causes a decrease of circulating testosterone levels (122). In 2018 and 2019, the Food and Drug Administration approved for patients with non-metastatic castration-resistant PCa apalutamide and darolutamide, respectively. These novel, effective and well tolerated AR antagonists tested in clinical trials, did not demonstrate the same efficacy on AR-positive BC patients (120). First and second generation of AR antagonists bicalutamide and enzalutamide, are the most used therapy for advanced BC, in particular in Tamoxifen-resistant and TNBCs (123, 124). They have been both used in clinical trials with positive results but still they are not used in the clinical practice (124) (**Table 2**). Most of the studies conducted with *in vitro* and *in vivo* experiments had the principal aim to test the dose, efficacy, safety, and tolerability of anti-AR therapies. A phase 1 study tested the anti-tumor activity of Seviteronel, a selective CYP17 lyase and AR inhibitor. The safety, tolerability, pharmacokinetics and activity of daily Seviteronel administration were evaluated in women with ER-positive tumors or TNBC and showed to be well tolerated (125). As Abiraterone acetate and CYP17A1 inhibitors, Seviteronel reduces the androgen production and is currently tested in phase 2 clinical trials (126) alone or combined with AR antagonists. However, both preclinical and clinical results indicated that AR in combination with other effectors fosters TN or HER2 positive BC growth (114, 117). Giovannelli P. and colleagues showed that in AR+ TNBC cell lines, S1 peptide could be a promising therapeutic option. In fact, it mimics AR proline-rich motif necessary for AR interaction with SH3-Src, leading to a reduced motility and invasiveness of TNBC cells (114). The *in vivo* experiments confirmed that S1 blocking could be a valuable anti-AR strategy. Lehmann’s group showed that AR enriched TNBC cell lines carrying PI3KCA mutations acquire sensitivity to PI3K/mTOR inhibition, promoting cancer cell growth (117). Some authors demonstrated that the combination of bicalutamide and PARP inhibitor (ABT-888) could inhibit cell viability and induce apoptosis in AR-positive TNBC (115). Regarding the correlation among AR, PARP1 and BRCA1 in TNBC, Sang et al. showed that AR and PARP1 expressions are negatively correlated with BRCA1 expression. Moreover, AR and PARP1 positively regulate each other in *in vitro* models (115). These findings suggest that the combination of bicalutamide and PARP inhibitors may be a potential strategy for TNBC patients. Krop et al. reported that the combination of low ESR1 and high AR expression identified a population of patients that seemed to have a benefit from the combination of enzalutamide and exemestane compared with exemestane alone [HR, 0.24 (95% CI, 0.10–0.60)] (116) (**Table 2**). Despite these recent advances in the knowledge of the involvement of AR signaling in TNBC, at present, there are no approved targeted therapies for these BC patients and robust data from prospective clinical trials are urgently desired.

## Conclusions

PCa studies suggest AR as prominent prognostic and predictive marker. However, the prognostic and predictive role of AR in BC is still matter of debate, since the results from clinical trials are not striking, probably due to both technical and biological reasons. Among the former, no companion diagnostic test to assess AR status and select eligible patients that could benefit from an anti-AR treatment is available. Disparities emerged in its evaluation due to different types of tests to detect AR, antibodies used, scoring systems and positivity cut offs. However, the prognostic role of AR expression detected by IHC and the ratio AR/ER in DCIS patients, could be worthy of further investigations. The differences in AR expression between primary and metastatic tumors suggest that AR should be detected in all patient biological materials, also considering the different role of this biomarker in the different subsets of disease. Although the real role of AR in predicting the response to endocrine therapy has to be defined yet, the ratios with hormone receptors should be taken into account, given their importance for patient risk assessment.

The possibility to treat AR positive TNBC patients with new anti-AR compounds, opens new perspectives in this prognostically unfavorable subset. However, additional studies are needed to verify the *in vivo* efficacy of the combination of anti-AR strategies.

## Author contributions

SB conceived the study. SR analyzed and interpreted the data. SR and SB were the major contributors in writing the manuscript. All authors read and approved the final manuscript.

## Funding

This work was partly supported thanks to the contribution of Ricerca Corrente by the Italian Ministry of Health within the research line 3, including genetics and environment in the development and progression of tumors and inhibitory mechanisms, exposomics and primary and secondary prevention.

## Conflict of interest

The authors declare that the research was conducted in the absence of any commercial or financial relationships that could be construed as a potential conflict of interest.

## Publisher’s note

All claims expressed in this article are solely those of the authors and do not necessarily represent those of their affiliated organizations, or those of the publisher, the editors and the reviewers. Any product that may be evaluated in this article, or claim that may be made by its manufacturer, is not guaranteed or endorsed by the publisher.
